# Leather Waste to Enhance Mechanical Performance of High-Density Polyethylene

**DOI:** 10.3390/polym12092016

**Published:** 2020-09-03

**Authors:** Eylem Kiliç, Quim Tarrés, Marc Delgado-Aguilar, Xavier Espinach, Pere Fullana-i-Palmer, Rita Puig

**Affiliations:** 1 Material Science and Nanotechnology Engineering Department, Usak University, 64200 Usak, Turkey; eylem.kilic@usak.edu.tr; 2 LEPAMAP Research Group, University of Girona, 17003 Girona, Spain; joaquimagusti.tarres@udg.edu; 3 Càtedra de Processos Industrials Sostenibles, University of Girona, 17003 Girona, Spain; 4 PRODIS Research Group, University of Girona, 17003 Girona, Spain; francisco.espinach@udg.edu; 5 UNESCO Chair in Life Cycle and Climate Change ESCI-UPF, Universitat Pompeu Fabra, 08003 Barcelona, Spain; pere.fullana@esci.upf.edu; 6 ABBU Research Group, Department of Computer Science and Industrial Engineering, Universitat de Lleida (UdL), 08700 Igualada, Spain

**Keywords:** buffing dust, HDPE, composites, recyclability, circular economy

## Abstract

Leather buffing dust (BF) is a waste from tannery which is usually disposed on landfills. The interest in using wastes as fillers or reinforcements for composites has raised recently due to environmental concerns. This study investigates the potential use of BF waste as filler for a high density polyethylene matrix (HDPE). A series of HDPE-BF composites, containing filler concentrations ranging from 20 to 50wt%, were formulated, injection molded and tested. The effect of filler contents on the mechanical properties of the composites were evaluated and discussed. Composites with BF contents up to 30wt% improved the tensile strength and Young’s modulus of the matrix, achieving similar mechanical properties to polypropylene (PP). In the case of flexural strength, it was found to be proportionally enhanced by increasing reinforcement content, maintaining high impact strength. These composites present great opportunities for PP application areas that require higher impact resistance. The materials were submitted to a series of closed-loop recycling cycles in order to assess their recyclability, being able to maintain better tensile strength than virgin HDPE after 5 cycles. The study develops new low-cost and sustainable composites by using a waste as composite filler.

## 1. Introduction

Leather industry converts raw skins or hides into stabilized leather. The transformation of 1000 kg of rawhide obtains only 200 kg of leather as final product, along with 250 kg of non-tanned and 200 kg of tanned waste [1]. About 25% of the tanned wastes are solid wastes from chromium-tanned leather. Shavings and buffing dust (BF) are within these wastes.

BF is a fine powder of collagenous waste containing chromium, synthetic fat and other chemicals. BF is generated when the leather is subjected to abrasion processes to achieve a uniform appearance. For every tone of skin or hide processed, about 2–6 kg of buffing dust is generated as solid waste [2]. Unlike chromium shavings, which can be converted into glue, gelatin [3] or collagen hydrolysate with appropriate treatments, the management of BF is difficult [4]. BF contains trivalent chromium, along with organic and inorganic compounds and may cause respiratory problems in humans exposed to environments containing BF particles [5]. Currently, these wastes are treated by incineration and/or landfilling. Both methods involve economic and environmental losses. Likewise, dumping of solid waste is subject to additional costs due to scarce availability of landfill sites and may account for soil and groundwater contamination due to leaching of chemicals into the landfill [2].

Circular economy strategies put pressure on the processing of wastes into secondary raw materials with the aim of their effective reuse instead of incineration or landfilling [6]. In this context, recycling waste materials as fillers for reinforcement of composites is growing interest. These inexpensive wastes can be used instead of virgin raw materials and can contribute to obtain composites with lower densities and higher mechanical properties. The literature shows some studies that replace raw materials with different types of leather waste for composites preparation. Some of these studies use chrome tanned leather wastes in the form of chromium shavings, which require multistage milling or grinding processes to prepare powder prior to its use [7]. Other researchers use BF without any additional or prior treatment [8]. Most of the papers refer to chromium shavings as fillers for different types of polymers (like natural fibers [9], newspaper fibers [10], cellulose [11], poly vinyl alcohol [10], polyvinylpyrrolidone [12], polyurethane [13], rubber [14], etc.) and fewer refer to BF. In the case of BF, its use together with rubber [15,16], polylactic acid [17], polycaprolactone [8], polystyrene [18] or epoxy-polymers [19] is described, usually to prepare low cost leather like materials suitable as footwear, clothing or construction.

The mostly contributing polymers to worldwide plastic consumption are polyvinyl chloride (PVC), polypropylene (PP) and high density polyethylene matrix (HDPE) [20]. In the case of packaging applications, the most commonly used thermoplastics are HDPE and polyethylene terephthalate (PET). In addition, post-consumer plastic packaging wastes need to achieve higher recycling percentages and higher quality of the recycled materials to be of industrial interest. Currently, post-consumer PET bottles are successfully sorted and recycled with high quality, thus putting HDPE into focus [21]. Therefore, the literature shows how different types of fillers have been incorporated into HDPE to produce low cost HDPE composites, obtaining materials with improved mechanical properties and less environmental impact [22,23,24]. Most of these composites use mineral fillers [25] or fillers from agricultural waste [26,27]. Unlike agroforestry waste fillers, fillers from leather industry waste are rarely reported. Earlier works used virgin and recycled low density polyethylene as polymer matrix for collagen hydrolysate (obtained from leather waste) [28,29,30]. The use of HDPE as polymer matrix was investigated only for chromium shaving, which is a different type of leather waste with higher fiber mean lengths, different structure and appearance [31].

In this work, BF filled HDPE composite materials were formulated, prepared and tested. Different BF contents, ranging from 20 to 50 wt%, were used to study the impact of such filler in the mechanical properties of the composites. Standard specimens were mold injected and tested under tensile, flexural and impact conditions. Interfacial adhesion and dispersion of buffing dust fibers in composite samples were also analyzed. The novelty of the present work is that, to the best knowledge of the authors, there is no literature about composites obtained from HDPE and BF. In the present study, leather-buffing dust was used as a filler to prepare low cost composites from leather waste fiber. This use avoids any additional pretreatment and finds an alternative use for BF, which is comparatively more difficult to manage than chromium shaving waste. HDPE is chosen as the polymer matrix due to its wide range of application areas such as packaging, pipes and tubes, wire and cable insulation, plastic lumber and more generally automotive and construction sectors [20].

## 2. Materials and Methods

### 2.1. Materials

Leather BF wastes were provided from a sheepskin processing leather factory in Turkey. HDPE Braskem 7252 was supplied by Nexeo Solutions (Sao Paulo, Brazil) and was used as thermoplastic matrix. A Fusabond E226 Maleic anhydride-grafted polyethylene (MAPE) by Dupont (Tamon-Carreño, Spain), was used as coupling agent to improve compatibility between fibers and HDPE.

### 2.2. Chemical and Morphological Characterization of Leather Buffing Dust

Prior to experimental studies BF was characterized in terms of moisture content, pH, chromium, fat, ash and nitrogen contents following standard procedures.

In order to determine moisture content, a weighed sample of BF waste was dried in an oven (ISO 4684). The pH value of BF was measured using 1:5 water extraction of samples by mechanical agitation for 6 h and subsequent determination with pH meter (ISO 4045). Total nitrogen values were determined with the Kjeldahl method (ISO 5397), with selenium catalyzed digestion of the waste sample. The determination of chromium oxide within BF waste was carried out by iodometric titration in aqueous solution (ISO 5398). The total ash content was analyzed by carbonization of the leather waste followed by treatment with sulfuric acid and ashing in an oven (ISO 4047). The fatty substance of BF waste was determined by solvent extraction method (ISO 4048). The measurement process was carried out for four samples.

Morphological analysis was carried out in a MorFi Compact analyzer (Techpap SAS, Grenoble France), equipped with a charged-coupled device (CCD) video camera. About 30,000 particles from a 25 mg/L suspension were analyzed by the MorFi v9.2 software. Among other parameters, mean fiber length, diameter and the amount of fine elements were determined.

### 2.3. Composite Preparation and Characterization

**Figure 1:**
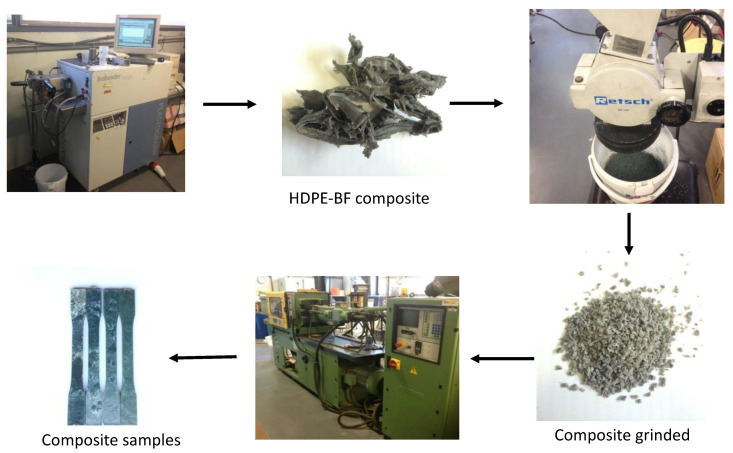
Composite samples preparation.

BF and HDPE pellets were dried at 80 °C, before processing, until constant weight. Composite samples containing 20, 30, 40 and 50 wt% of BF were prepared in a Brabender^®^ internal mixing machine (Figure 1). A sample with 30 wt% BF and 6% MAPE contents was also prepared. MAPE percentage was based on the available literature and the research group’s experience [32].

For some experiments, leather BF was degreased following modified ISO 14048 standard by means of suspending it on dichloromethane for three hours under gentle stirring. The suspension was permanently heated and kept at the boiling point of the organic solvent.

In all cases, materials were compounded at 80 rpm speed for 10 min at 170 °C. Thereafter, the blends were cut down into pellets in a blade mill with a 10 mm mesh and kept in oven at 80 °C until further use to prevent moisture absorption. The composites were injection molded in a Meteor 40 injection-molding machine (Mateu & Sole, Spain) to produce test specimens complying with ASTM D3641 specifications (see Figure 1).

The composite specimens were placed in a conditioning chamber (Dycometal) at 23 °C and 50% relative humidity for 48 h, prior to mechanical testing in accordance with ASTM D638. Tensile tests of specimens were carried out on an Instron TM112 testing machine (Norwood, MA, USA), fitted with a 5 kN load cell, operating at a rate of 2 mm/min in accordance with ASTM D638 standard. Results were obtained from the average of at least 5 composite samples. Young’s modulus was analyzed with an extensometer.

Flexural tests were performed according to standard ASTM D790, using the same dynamometer used for the tensile strength but equipped with three-point bending clamps. The absorbed energy under impact conditions was determined for notched and un-notched Charpy impact test, following the ISO 179 standard. A Resil 5.5 hammer, supplied by Ceast (Norwood, MA, USA), was used for measurement. From the ten-specimen experimental batch, five were notched in order to obtain the energy for fracture propagation. The other five samples were tested as injected, aiming at measuring both fracture generation and propagation.

Some already-tested specimens were used for Scanning Electron Microscopy (SEM) analysis, aiming at observing the interphase between the filler and the matrix. A Hitachi S-3000 variable pressure SEM microscope was used. The acceleration voltage was 7.0 kV.

### 2.4. Recyclability Test of Composite Specimens

The suitability of the obtained composites to be recycled was also assessed through performing multiple recycling cycles. Recyclability of the obtained composites was determined by means of submitting the reinforced composite test specimens to milling, injection-molding process followed by testing the recycled specimens for tensile strength at break, Young’s modulus and elongation at break. Recycling process was repeated until obtaining a tensile strength at break value equal to virgin HDPE. Neat HDPE was also submitted to the recyclability tests in order to quantify the strength loss due to recycling.

## 3. Results and Discussion

It is well known that some mechanical properties of composite materials depend on factors, such as (i) the original properties of the matrix, (ii) the intrinsic properties of the reinforcement, (iii) the dispersion and orientation of the reinforcement, (iv) the interphase between both phases (v) the morphology of the reinforcement, mainly its aspect ratio and (vi) the reinforcement ratio in the composite [33]. In fact, the chemical composition of the reinforcement and of the matrix have a crucial role on the interphase between phases and the dispersion of the reinforcement. In this sense, the chemical composition of BF was determined, aiming at further understanding the interactions that may take place (Table 1).

The main compound in BF was found to be collagen protein, as expected. Considering the previously reported values by several authors [8,17,34] for ash, protein, nitrogen, chromium oxide content and pH values within BF, the values measured in our study were well within the limits, except the content of moisture and fatty substance. In our work the fatty substance rate (7.9%) in BF was found to be significantly higher than the previously published value, which was only reported by Reference [34] as 1.97%, this might have some effect on the compatibility between HDPE and BF. Taking into account the high water content of BF, both BF and HDPE were dried in an oven until constant weight, aiming at minimizing the risk of imperfections inside the composite and, thus, the generation of weak points from where fracture may easily initiate under stress. This high water content indicates the hydrophilicity of the protein, which, at the same time, can be limited by the presence of fatty substances. Such fatty substances, indeed, can promote the reinforcement dispersion due to its lubricating effect, fostering the flow and free movement of BF during compounding.

For all the above, two different strategies were adopted. On the one hand, due to the high protein content of BF, the use of a coupling agent based on MAPE was considered, since it has been reported to be advantageous for HDPE composites reinforced with hydrophilic phases [37]. On the other hand, degreasing BF to further increase its hydrophilic character thereof, aiming at promoting the interactions between BF, HDPE and, if appropriate, MAPE.

### 3.1. Effect of MAPE and Degreasing BF on the Mechanical Properties of the Composite

**Figure 2:**
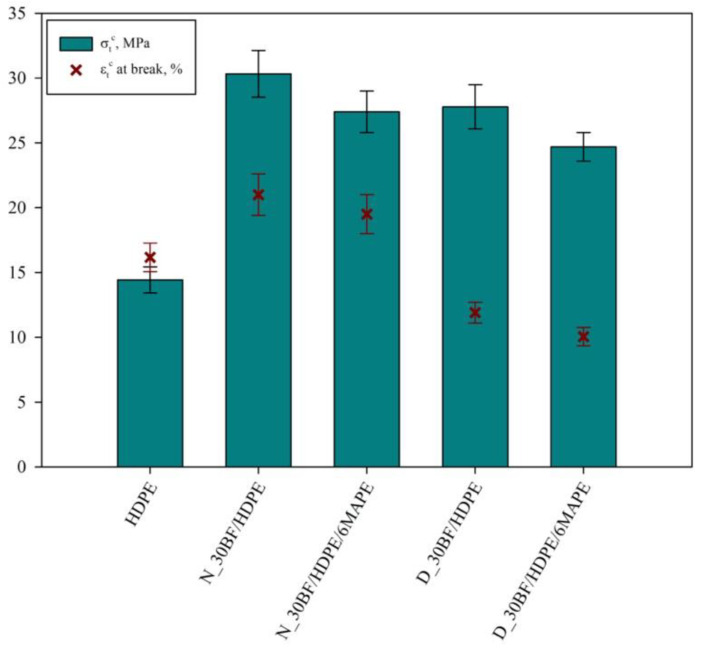
Tensile strength and elongation at break of 30 wt%-reinforced high density polyethylene (HDPE) composites. N_30BF and D_30BF are referred to neat and degreased buffing dust (BF), respectively.

**Figure 3:**
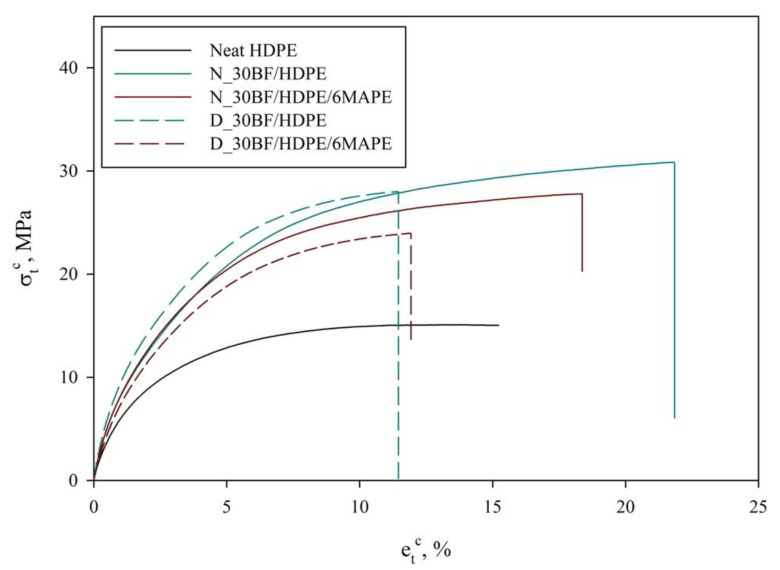
Stress–strain curves of the obtained materials.

With the purpose of glimpsing the effect of MAPE and fatty substances, composites containing 0 and 6 wt% of MAPE and reinforced with a 30 wt% of either neat or degreased BF were prepared and tested for tensile strength and elongation at break (Figure 2). Values presented in Figure 2 are average values obtained from 5 different specimens. In Figure 3, stress–strain curves of the obtained composites are shown for the most representative specimen of each composite.

Neat HDPE exhibited a tensile strength at break of about 14.43 MPa and an elongation at break of 16.17%. When 30 wt% of neat BF was incorporated into the HDPE matrix, it was found that the tensile strength at break was improved doubling its value (from 14.43 to 30.33 MPa). Surprisingly, the elongation at break, instead of decreasing, increased up to 21.00%. This is completely atypical for short fiber reinforced polyolefin, at least with those made of carbon, glass, aramid or even natural fibers [33]. Usually, the composites reduce its strain at break due the inclusion of a more fragile phase.

In this sense, apparently, the use of BF is beneficial in terms of improving the mechanical strength of the material, as well as its elongation at break and, thus, its ductility. Several studies have reported the negative effects of collagen when reinforcing thermoplastic polymers, especially in terms of mechanical strength and Young’s modulus. However, in this case, the authors were not incorporating only collagen but also other components such as synthetic fat and other chemicals that might contribute to the enhancement of tensile strength. The presence of collagen was enough to increase the ductility of the composite materials, at least with the presence of 30 wt% of BF. In fact, this ductility will be later corroborated by means of impact strength assessment for notched and un-notched specimens.

On the other hand, when degreased BF was incorporated into the HDPE, tensile strength at break was enhanced in a 92.5%, achieving a value of 27.78 MPa. The obtained value was slightly lower than the one from the N_30BF/HDPE sample, making the degreasing process unjustifiable, even when 6 wt% of MAPE was added to the composite (see Figure 2). Experiments with degreased BF show the typical behavior of fiber-reinforced thermoplastic composites, where mechanical performance is enhanced, while ductility and deformability are negatively affected [38].

Aiming at even improving the mechanical performance of BF reinforced HDPE composites; MAPE was incorporated at a ratio of 6 wt% referred to HDPE content. MAPE had not any significant effect on those composites containing neat BF nor degreased BF. This was expected in the case of neat BF as it is already hydrophobic but in the case of degreased BF a noticeable effect was expected, due to its hydrophilic character. Nonetheless, in both cases, tensile strength was slightly decreased and elongation at break remained almost constant. Apparently, the maleic anhydride rings in MAPE should create hydrogen bonds between HDPE and the protein in BF, as in the case of glass or cellulosic fibers [39,40]. However, it becomes apparent that the rest of compounds present in the BF, such as ash or chrome oxides, limited these interactions. For all the above, no MAPE was used for the rest of composites.

### 3.2. Mechanical Properties of HDPE Composite with Different Proportions of BF

**Figure 4:**
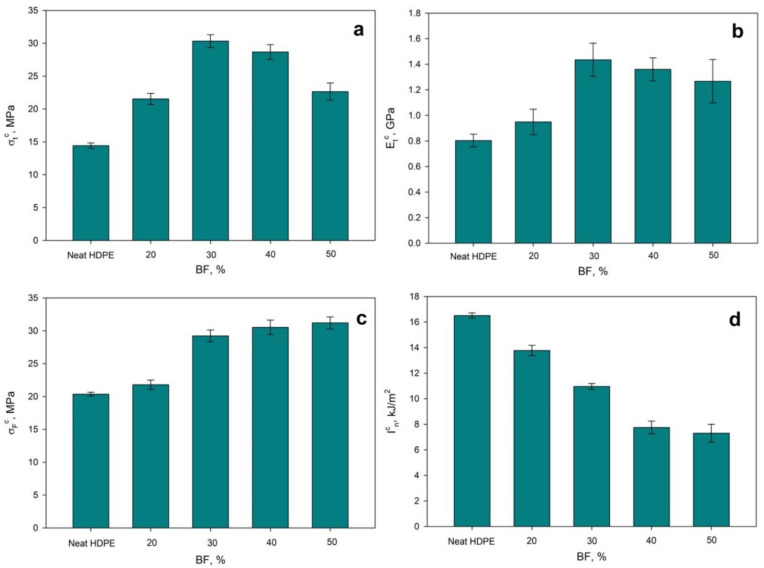
Tensile strength at break (**a**) Young’s modulus; (**b**) flexural strength; (**c**) and impact strength (notched); (**d**) of BF reinforced HDPE composites.

HDPE composites with 0% to 50% BF were prepared and characterized in terms of tensile and flexural strength, Young’s modulus and impact strength. Figure 4 shows the obtained results as function of the BF content.

#### 3.2.1. Tensile Strength at Break and Young’s Modulus

As observed in Figure 4, the incorporation of BF had a positive effect on the tensile strength of HDPE composites up to 30 wt% of reinforcement content. However, higher contents of BF slightly decreased the tensile strength. This was attributed to BF agglomeration or bad dispersion, which probably resulted in weak points for fracture initiation [34] and poor interphase between both constituents as well. The same behavior was observed for the Young’s modulus.

Considering that collagen protein due to its triple helix structure has a noticeable impact in the tensile strength of bones, teeth, cartilage, tendon, fibrous matrices and elasticity to skin, the evolution of tensile strength observed in the BF-based composites may be attributed to collagen’s characteristics [41]. Providing that the inherent fibrous nature of leather waste is retained during composite processing, it could function as reinforcement for the matrix [42]. Nonetheless, Ramaraj [43], found that with moderate addition of buffing leather waste to an ABS matrix, the mechanical properties were enhanced until certain point, to be later decreased, similarly to what has happened in the present study.

Something interesting is that those composites with 30 wt% reinforcement exhibited a similar tensile strength at break to polypropylene (PP), as well as a similar Young’s modulus [43]. Taking into account the obtained tensile strength and modulus, one could expect lower elongation at break and, in addition, lower impact strength of notched specimens than the values shown in Figure 4d. In fact, the impact strength of notched specimens made of PP usually exhibit an impact strength of about 4 kJ/m^2^, thus lower than the value achieved with the HDPE/BF composites [44]. In this sense, the proposed composite materials offer a great opportunity on those applications where the mechanical performance of PP is required but the product is exposed to impacts. Notched specimens were used to measure the energy needed to propagate a crack. In fact, the crack usually tends to propagate through the weakest phase in a composite material, either matrix, filler or interphase [45]. According to the results, the interphase is the weakest phase (see the negative evolution of impact strength as the amount of filler is increased).

#### 3.2.2. Flexural Strength

Regarding flexural strength (Figure 4), it was found to be enhanced as the amount of BF was increased. Usually, flexural strength exhibits a similar behavior than tensile strength, as it has been previously reported [46]. Nonetheless, flexural strength is also strongly affected by compression strength, fact that could cause the different behavior. Nevertheless, considering the standard deviation of the tested specimens, it can be stated that there were no significant differences between those composites reinforced with 30 wt% of BF and those with 50 wt%. In any case, the flexural strength of the 30 wt% reinforced composite was slightly lower than the one of PP [33].

#### 3.2.3. Impact Strength

During Charpy test there are two main phenomena related with the impact strength. On the one hand, the energy required to create a fracture and, on the other, the energy needed to propagate such fracture. The energy for propagating the fracture can be considered as the joint effect of the energy required to break the fiber, the energy required to break the matrix and the energy required to break the interphase between BF and HDPE, like BF sliding or pullout [47]. Thus, the notched Charpy test corresponds to the propagation of the fracture (involving the three abovementioned effects) and the unnotched Charpy test involves both the energy required to create the fracture and the one to propagate it. In this sense, the difference between both tests reveals the energy required to initiate the fracture. However, the only specimen that broke when submitted to the unnotched Charpy test was the one reinforced with 50 wt% of BF, exhibiting an impact strength of 39.66 kJ/m^2^. The results bring to the light that the obtained materials present great opportunities in those applications where high impact resistance is required and the tensile characteristics of polypropylene, for instance, are enough. In fact, there are few applications where materials are submitted to tensile stresses, while flexural and impact are the most abundant [48]. It must be mentioned that as the amount of BF was increased, impact strength decreased (Figure 4d). This can only be explained, again, by the weak interphase between BF and HDPE.

### 3.3. SEM Images of BF-Reinforced HDPE Composite

**Figure 5:**
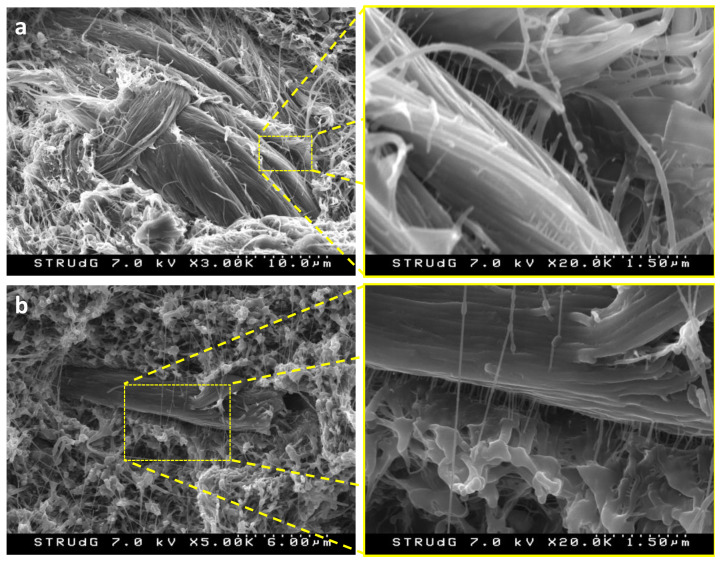
Scanning electron microscopy (SEM) images of 30 wt% (**a**) and 50 wt%; (**b**) BF-reinforced HDPE composites at different magnification.

The broken specimens were observed by means of scanning electron microscopy (SEM) (Figure 5). The SEM images at low magnification reveal that BF structures were not homogeneously dispersed within the matrix, as relatively big particles can be observed. This is in accordance with what was observed before—tensile strength and Young’s modulus did not evolve linearly with the amount of BF, indicating that dispersion of the reinforcement was poorly achieved. However, Figure 5 also reveals that some fibrillar-like interactions were obtained between BF and HDPE, especially observed in those images at higher magnification (×20).

### 3.4. Recyclability Test of the Composite

**Figure 6:**
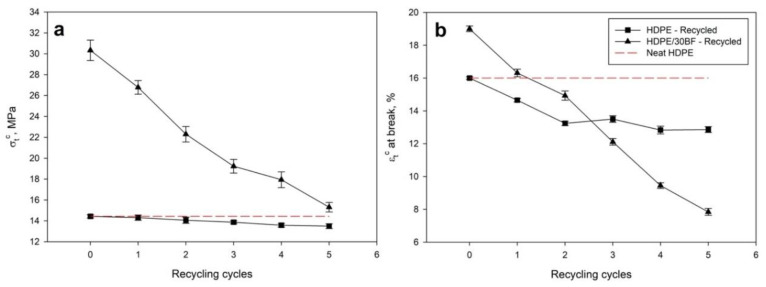
Effect of successive recycling cycles on tensile strength at break (**a**) and elongation at break; (**b**) of HDPE and HDPE composites.

For the recyclability test, the 30 wt% reinforced composite was submitted to several milling and mold injection cycles, aiming at determining mechanical property losses during recycling. In parallel, HDPE specimens were also submitted to the same recycling processes. Results for elongation at break and tensile strength at break after each recycling cycle are given in Figure 6.

It was observed that elongation at break and tensile strength at break of neat HDPE decreased as the number of recycling cycles increased. This loss of properties can come from several effects, being the most important those related to shortening of polymeric chains and, thus, reduction on molecular weight, increased entanglement of polymer chains or the creation of cyclic compounds due to reactions between side chains. The plastic recycling is clearly justified by economic and environmental reasons. On the one hand, the use of recycled materials palliates the inflation on prices of virgin raw materials. On the other hand, the use of recycled materials implies less energy consumption [49], independence or lower dependence from oil and reduces the amount of landfilled oil-based materials [50]. In addition, recycling plastic materials by preparing composites containing other materials, such as the case of BF, contributes to the control and inertization of residues with potential risk to the environment and human health. However, as it is reflected in Figure 6, the relative property loss is even higher than when neat HDPE is used. Nonetheless, Figure 6 brings to the light that those HDPE composites containing 30 wt% of BF could be recycled up to 5 times, while exhibiting higher tensile strength at break than neat HDPE. However, only one recycling cycle is required to achieve the same elongation at break that neat HDPE, being considerably decreased in the following cycles. This effect is crucial and deserves special attention, since it is a clear indication of the fragility of the material.

In fact, this decrease on mechanical properties is mainly due to the changes that BF experienced in terms of morphology. Indeed, Table 2 shows the differences on average length, diameter and other morphological parameters of BF, before and after compounding (Cycle 0).

**Figure 7:**
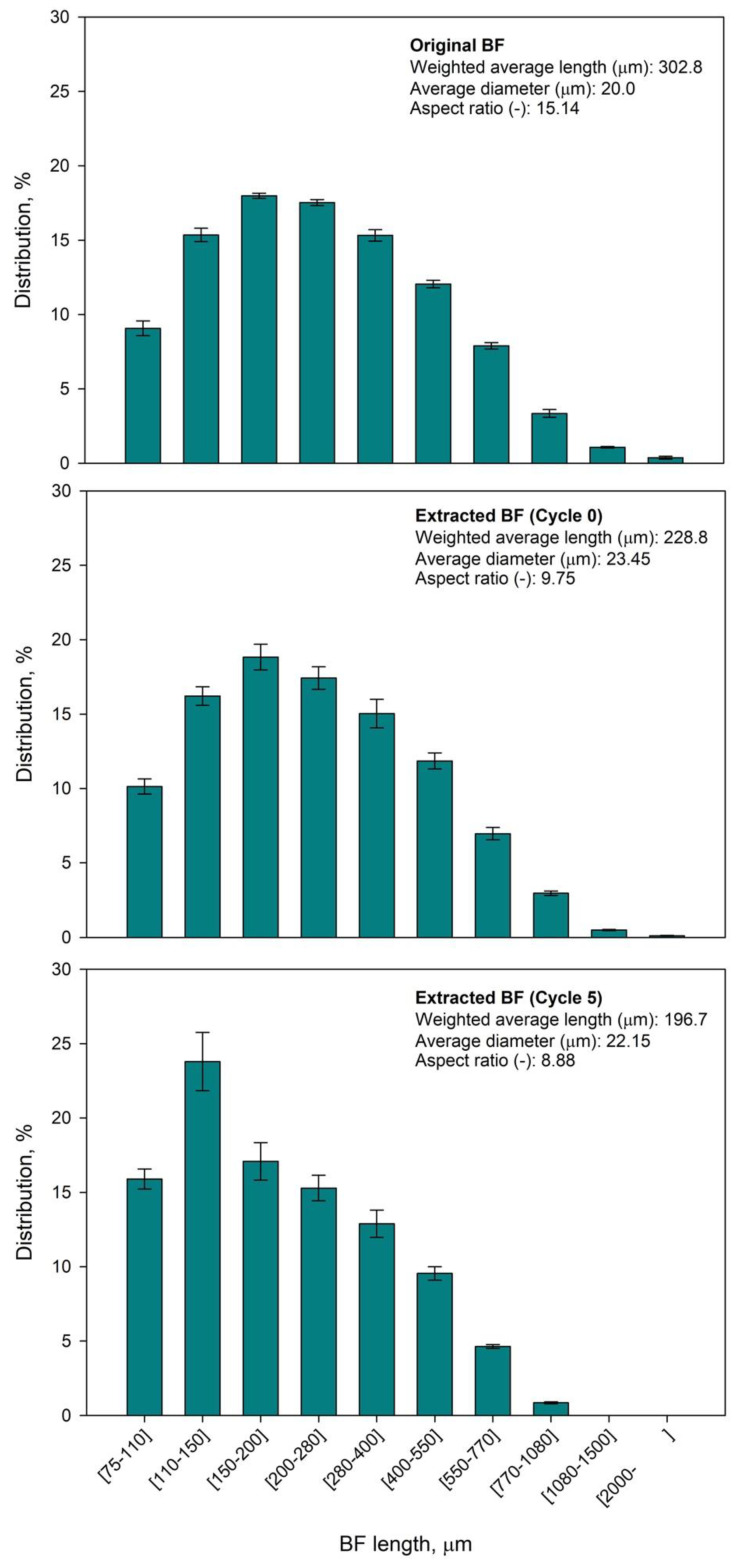
Fiber length distributions of the original BF and the extracted BF after Cycle 0 and Cycle 5.

As can be seen in Table 2, both the compounding and injection molding processes significantly affected the length of the BF fiber. This is in line with the results that have been already reported by several authors in the case of lignocellulosic fibers, where the effects of processing the composites negatively affect the potential mechanical performance of the material [33,51], influencing as a rebound effect the environmental performance [52]. Indeed, when comparing the fiber length distributions of the extracted BF after the cycle 0 with the one from cycle 5, a clear shift to the left of the graph can be observed (Figure 7).

In addition, the aspect ratio, understood as the ratio between length and diameter, was decreased as the composites were further recycled. This can be explained by the fact that BF became shorter as it was submitted to compounding and injection molding processes, while its diameter remained almost constant. This decrease on aspect ratio may affect the reinforcing potential of BF in HDPE composites, as it has been extensively reported [53]. Thus, the slenderness of the reinforcement will impart an important role on the ultimate mechanical properties of composites, mainly due to the available surface area to bond the matrix and the capacity of creating a network throughout the material [53].

### 3.5. Summary of the Main Results Obtained

As a summary, the addition of 30% BF to HDPE matrix improves its mechanical properties (specifically tensile strength at break, flexural strength and Young’s modulus about 110%, 75% and 50% improvement respectively) with also higher impact strength (as unnotched specimens did not break with Charpy test). This gives a composite with similar tensile and Young’s modulus properties than PP and higher impact strength, very promising for applications where the mechanical performance of PP is required but the product is exposed to impacts (for example, car bumpers). In addition, this composite could be recycled up to 5 times, while exhibiting higher tensile strength at break than neat HDPE.

Addition of 30% BF to recycled HDPE could be also a promising option to enhance the mechanical properties downgraded during the recycling process. The quality of recycled thermoplastics is lower as its polymer chain grows shorter during the recycling process [54]. The addition of BF might help to achieve better mechanical properties to be able to use recycled HDPE thermoplastic for higher value applications instead of the actual downcycling options [55], thus increasing the circularity of HDPE.

## 4. Conclusions

A composite material made out of HDPE and BF (a waste from leather making) was produced by melt mixing and injection molding. The mechanical characterization of this composite was performed my means of tensile, impact and flexural properties to determine the effect of BF fiber addition on the mechanical properties of the polymer matrix. Flexural strength, tensile strength and Young’s modulus of the tested composites enhanced as the percentage of the reinforcement material increased up to 30%, while the last two properties slightly decreased at higher percentages of BF. These results were supported by SEM images, in which poor dispersion and agglomeration of fibers were observed at higher BF contents. In addition, HDPE-BF 30% composites presented noticeable impact strength, as unnotched specimens did not break. Composite samples achieved successful recycling cycles up to five times and maintained the tensile strength at break above that for a virgin HDPE sample. These composite materials are a promising alternative in applications where high impact resistance is needed together with the tensile characteristics of PP (i.e., car bumper).

BF waste showed to be a promising reinforcement for HDPE-based composites, not only with good mechanical properties but also with a lower cost and high strengthening capabilities. The current study presents a good opportunity to reduce the environmental impact of leather production by re-valorizing a leather production waste (BF), through reuse and converting it into an added value product, as an alternative to landfilling or incineration. In addition, the studied composite material can help increasing circularity of HDPE thermoplastic.

## Figures and Tables

**Table 1:** Chemical composition of buffing dust (BF) waste.

Tests	Values	Reference Data	
Ash (wt%)	12.1	12.3%, 12.86%, 12.86%, 12.86%	[8,17,34,35]
Chromium oxide (wt%)	4.7	3.14%, 3.41%, 3.41%	[8,17,34]
Fatty substances (wt%)	7.9	1.97%	[34]
Nitrogen (wt%)	10.3	7.03%, 9.71%, 9.71%	[17,34,36]
Protein (wt%)	57.5	54.58%, 54.58%	[17,34]
Moisture (wt%)	50.9	14%, 7.9%, 7.92%, 7.92%	[8,17,34,35]
pH	5.25	3.8, 4.15, 4.5	[8,17,35]

**Table 2:** Morphological analysis of original and extracted BF from 30 wt% reinforced high density polyethylene (HDPE) composite.

	Length Weighted in Length (µm)	Diameter (µm)	Fine Elements (%)
Original BF	302.8 ± 1.2	19.98 ± 0.13	50.7 ± 0.7
Extracted BF	228.8 ± 12.5	23.45 ± 0.40	66.8 ± 2.9
